# I Don’t Really Know Much About PrEP: Examining Black HBCU Women’s Pre-Exposure Prophylaxis Knowledge and Awareness

**DOI:** 10.3390/ijerph22121813

**Published:** 2025-12-03

**Authors:** Marissa N. Robinson, Brittany M. Williams, Gloria Aidoo-Frimpong, Reginald A. Blockett, Calvin R. Lowery, Michelle Sandoval-Rosario, Rasheeta Chandler

**Affiliations:** 1 Department of Global Health, Milken Institute School of Public Health, George Washington University, Washington, DC 20053, USA; 2 Department of Education, University of Vermont, Burlington, VT 05405, USA; brittany.williams@uvm.edu; 3 Department of Epidemiology and Environmental Health, University at Buffalo, Buffalo, NY 14214, USA; gloriaai@buffalo.edu; 4 Department of Educational Foundations, Leadership, and Technology, Auburn University, Auburn, AL 38649, USA; rzb0118@auburn.edu; 5 Health and Human Sciences Advising Center, University of North Carolina at Greensboro, Greensboro, NC 27412, USA; crlowery@uncg.edu; 6 Independent Researcher, Washington, DC 20201, USA; msandoval1224@gmail.com; 7 Nell Hodgson Woodruff School of Nursing, Emory University, Atlanta, GA 30322, USA; r.d.chandler@emory.edu

**Keywords:** black women, college students, HBCUs, HIV, pre-exposure prophylaxis (PrEP), HIV prevention

## Abstract

Despite HIV/AIDS disproportionately impacting Black women in the United States, HIV knowledge, awareness, and uptake of prevention efforts like pre-exposure prophylaxis (PrEP) have been limited for this community. Since campus leaders can maintain the attention of Black college women for a sustained period, they are an ideal demographic for PrEP interventions. Accordingly, the purpose of this study was to assess the awareness and knowledge of PrEP among Black Historically Black Colleges and University (HBCU) women, informing future possibilities and strategies for PrEP interventions and uptake. The lead author employed a descriptive, qualitative approach to collect data from five focus groups of Black HBCU women. Within this, the authors examined Black HBCU women’s PrEP knowledge and awareness in their own words and on their own terms. The results revealed that Black HBCU women have notable gaps in knowledge and limited awareness about PrEP and sexual health, with some assuming PrEP is only for men who have sex with men (MSM). Others believed it was used to manage an existing HIV diagnosis. Given that these participants perceived PrEP as primarily for MSMs, there is a clear gap in public health practice. Therefore, it is essential to focus future HIV prevention efforts on college-aged Black women by exploring their perspectives and considering how institutions like student activities, Greek Life, and campus health services can contribute to PrEP education for HIV prevention.

## 1. Introduction

Approximately 1.2 million Americans are living with Human immunodeficiency virus (HIV), while 13% are unaware of their condition [1]. In 2021, estimates indicated about 32,100 new cases of HIV were reported annually within the United States; however, cisgender Black women are disproportionately impacted and accounted for approximately 57% of new HIV diagnoses despite being only 13% of the female population [1,2]. Prevalence of HIV is high in the Southern US, with over 52% of new cases annually [1]. There are evident disparities among women living with HIV based on characteristics such as race, location, shame, stigma, and socioeconomic inequalities, including health literacy, being uninsured, and lack of quality healthcare access [1,3,4,5,6]. Numerous factors, including high HIV prevalence within sexual networks, sexual partner concurrency, imbalanced male-to-female sexual partner ratios, partner instability, and low relative frequency of consistent or correct condom use, can increase the odds of exposure to HIV among women [7,8].

Although Black women are disproportionately impacted and overburdened by HIV acquisitions due to multiple social, structural, and systemic factors, pre-exposure prophylaxis (PrEP), a once-daily pill or long-acting injectable to prevent HIV transmission—the advanced and effective biomedical intervention—is not widely used among Black women who can potentially benefit from it [9,10,11,12]. Researchers recommend combining PrEP with other preventive sexual practices such as condoms to significantly reduce the likelihood of acquiring HIV and other sexually transmitted infections (STIs) through sexual contact [9,12,13]. And while the CDC estimated 400,000 Black women in the United States could benefit from PrEP, some data suggest only 3% with a clinical indication were prescribed the prevention medication by their healthcare provider [14]. This poses a problem because the bulk of the extant literature focuses on MSM in relation to PrEP, while failing to consider the differences among Black men and women [15,16,17,18,19,20]. Such normalities further reduce Black women’s visibility within and possible viability for PrEP uptake.

The World Health Organization estimates nearly 1 million cases of STIs (chlamydia, gonorrhea, trichomoniasis, and syphilis) occur daily around the world [21]. Comparatively, 20 million STI cases are reported in the US annually, and more than half occur among those of traditional college-age (ages 13–24) [22,23]. According to the CDC’s updated PrEP guidelines, bacterial STIs, including chlamydia and gonorrhea diagnoses, serve as clinical indications for PrEP initiation [24]. However, provider awareness of these guidelines remains limited, and PrEP prescribing practices are inconsistent, even among populations with the highest adherence rates, such as MSMs, signaling that they are likely worse for Black women [25,26]. Nonetheless, while research on Black women and PrEP is increasing, there remains a need for additional data regarding the knowledge and awareness of PrEP among Black cisgender women in the college context, and especially those attending historically Black colleges and universities (HBCUs) [9,27,28,29]. This is partly because the federal government, researchers, and CDC do not engage in federal tracking of HIV status and student enrollment status, but also because even though Black college women often have higher HIV knowledge than their white peers, they frequently underestimate their personal HIV risk and exhibit low PrEP awareness and uptake [19,20,29].

Previously, scholars have explored how and why U.S. colleges and universities are integral sites for PrEP-related interventions and uptake among Black college women [19,20]. They have also examined Black college women’s PrEP perceptions and reception to PrEP-related content delivery [28], and considered barriers and facilitators within Black college women’s PrEP decision-making [19]. Across these studies, some Black college students have demonstrated they are knowledgeable of HIV, the modes of its transmission, and strategies to prevent exposure to it [9,30,31,32]. However, this knowledge does not consistently translate into behaviors that prevent the sexual transmission of HIV. Relatedly, many college students continue to underestimate their risk of acquiring HIV and other STIs, as the data on these realities are inconsistent and often unknown [33,34].

Within the existing research on women and PrEP broadly, researchers agree that women are strongly interested in using PrEP once they learn about it [35]. In fact, PrEP has been identified as a vital women-controlled HIV prevention mechanism [9], unlike condom usage or the withdrawal method, which both tend to be male-controlled and centered. However, information surrounding PrEP for HIV prevention strategies among students attending HBCUs is limited [31,36,37], as is a broader focus on cisgender women and PrEP [38,39]. More than this, of the existing research on HBCUs and PrEP, much of the PrEP promotion literature center on organizational readiness, implementation outcomes, and early PrEP explorations on students’ likely PrEP acceptance [40]. It is important to consider the HBCU context given the proportion of Black college women they educate, and the unique experiences Black students report because of the HBCU environment [41]. Moreover, since HBCUs exist across a range of geographical contexts (i.e., both urban and rural), and geographies are associated with HIV testing, prevention, and treatment uptake, they are a vital context to explore regarding HIV prevention modalities [42,43].

Indeed, HBCUs are “living ecosystems of genius… wherein culture becomes curriculum, where resilience becomes research, and where access becomes transformation” [44]. Moreover, there is existing evidence to suggest that legislative and policy changes for HBCUs have had (un)intentional positive impacts on predominantly white institutions and students [45]. If we hold this to be true, then the downstream implications of PrEP uptake and programming in HBCU environments may also have a similar impact across higher education more broadly [40]. These realities underscore the importance and significance of PrEP interventions, studies, and explorations that are site, context, and culturally significant, and that account for the range of experiences Black women have [39,40,41,42,43,44,45,46,47,48]. Accordingly, the purpose of the present study was to explore heterosexual, cisgender Black college women’s PrEP knowledge and awareness while enrolled at an HBCU. Using a qualitative descriptive approach, the following question guided this study: “What is Black HBCU college women’s PrEP awareness?”

## 2. Methods and Approaches

The present study builds upon the previous research conducted by Chandler et al. regarding PrEP utilization among college-aged Black women [28]. Chandler et al. broadly explored Black college women’s awareness and perceptions of PrEP use and preferences for information delivery using the health belief model as the theoretical framework [28]. Their findings suggested that Black college women were open to considering PrEP when provided a PrEP-inclusive education intervention. However, the study largely focused on the modality of PrEP information delivery rather than recognizing that most participants had never heard of PrEP or considered themselves potential candidates for its uptake. Accordingly, the authors extended the scope of the parent study in a larger project to investigate additional contextual factors beyond individual characteristics to gain a deeper understanding of Black college women’s reported gaps in PrEP knowledge and awareness. These factors include attitudes towards PrEP uptake, historical traumas related to public health research, HIV-risk beliefs, and perceptions all within the HBCU context.

The present study draws from a larger inquiry building upon Chandler et al.’s initial Black college women and PrEP scholarly inquiry [28]. To achieve this aim, the authors used a basic interpretive qualitative approach, operationalizing virtual focus group methods [49,50]. Basic interpretive qualitative designs are useful in contexts where little context and data exist around a specific topic, but there is an urgent need to understand, interpret, and illuminate participants’ perspectives about the subject [38]. Since there is a dearth of research on Black college women and PrEP in HBCU contexts [19], this was an ideal strategy to generate the fullest possible data. The authors intentionally leveraged qualitative focus group design in the present study because focus groups provide vital insights for scholars to contextualize participant responses within the social contexts they negotiate [51,52]. Said plainly, students’ likelihood, values, and articulated conceptions around PrEP are informed by their peers. Therefore, considering their answers and choices within a peer-to-peer [53] dynamic was vital to understanding PrEP knowledge and awareness in context.

Since the 1950s, focus groups have been held as a key strategy for better understanding how individual and collective group dynamics shape interpersonal decision-making [50,51]. While PrEP uptake is an individual decision, PrEP awareness and knowledge are deeply tied to cultural and social factors [52,54]. More than this, Black women’s unique communication praxis and approaches to individual health choices are sometimes informed by communal dialogue in group settings [54,55]. Ultimately, the authors positioned the focus groups approach as a time-effective research strategy, making it appropriate for the present study as it allowed them to gather richer, more in-depth data [52,56]. The focus group guide was developed using the health belief model framework with questions initially conceived of by Chandler et al., which established participants’ beliefs and attitudes about PrEP [28].

### 2.1. Recruitment and Data Generation

After receiving Institutional Review Board (IRB) approval for all study procedures, the lead author recruited participants from January 2022 to March 2022. The lead author used word of mouth and electronic flyer distribution among Northeastern HBCU students. The eligibility criteria was: (a) self-identified as Black/African American, (b) assigned female at birth, (c) currently enrolled at the Northeastern HBCU, (d) was 18 years or older and actively sexually engaged (including but not limited to manual stimulation, dry humping, digital stimulation, penetrative anally, orally, vaginally, or experimentally), (e) HIV negative, and (f) had multiple sexual partners and/or had inconsistent condom usage. This criterion for inclusion was necessitated by the aims of the study. A total of 200 prospective participants indicated interest in the study, of whom 138 participants completed a prescreening inclusion criteria survey via Google Form to determine eligibility.

Following eligibility determination, 40 eligible participants were contacted by their preferred method of choice (text, phone, or email) and were provided an informed consent form to confirm their participation. A total of 22 participants formally enrolled and completed the study. After enrollment, participants completed an online scheduling survey to identify suitable times for 60 min virtual focus group discussions (FGDs). In total, five FGDs were conducted with 22 college women participating. During these audio-recorded FGDs, the lead author guided participants through a semi-structured focus group using a moderator guide. Direct questions were asked, including (a) What type of information do you think you and/or women like you need to know about sex and reproductive health?; and (b) What have you heard about PrEP? Participants were compensated $25.00 USD through an electronic gift card. The lead author conducted and transcribed each focus group, subsequently adding all additional authors to help with the final manuscript completion upon conclusion of data analysis.

### 2.2. Participant Characteristics and Data Analysis

In Table 1, the authors provide an overview of the demographic characteristics reported to us by all participants. The majority of the participants were aged between 18 and 25 years (86.4%). Freshmen constituted 45.5% of the participants, and 63.6% reported being single. Although all participants met the study eligibility criteria of having multiple sexual partners or reporting inconsistent condom use in the recent past, approximately 86.4% of the students reported not having multiple sexual partners at the time of data collection, and half reported consistent condom use. Most participants identified as undergraduates (86.4%), some students were enrolled at their HBCU for graduate education (doctoral level; 13.6%).

To analyze the FGD data and move beyond self-reported demographics, the authors used Braun and Clarke’s six-step method for data analysis, which includes: (1) selection of statements; (2) selection of keywords; (3) coding; (4) theme development; (5) conceptualization; and (6) conceptual framework development [57]. First, the transcripts were reviewed and organized by the principal investigator, making notes and starting to categorize personal shared reflections into keywords. Next, the authors generated MAXQDA 2022 software codes to help identify similarities and differences among the categories developed manually. The software clustered some responses together due to similarity. Then, single-word descriptors were identified from the data to begin the process of developing themes. For example, situationship and sneaky link were initially separately categorized, but eventually collapsed into not monogamous because of their congruence. Said plainly, the further subcategories culminated into major categories, which were named and defined to address the research question. To ensure confirmability, the authors employed various strategies, including member checking, audit trails, thick descriptions, and an analysis codebook [56].

## 3. Results

Overall, the findings revealed significant knowledge gaps and limited awareness concerning PrEP specifically and sexual health generally among Black college women at an HBCU. Three key themes emerged from this data: (1) limited knowledge of PrEP; (2) the need for comprehensive sex education; and (3) possibilities and potential for PrEP as a long-term strategy. These findings align with prior qualitative research among Black women at other HBCUs [19,28], and highlight contextual factors such as campus culture, access to sexual health resources, historical medical mistrust, and prior gaps in sex education. Consistent with previous studies, Black women also demonstrated low awareness of PrEP and nuanced perceptions of PrEP as an HIV prevention tool, which may influence uptake [31,34]. In the first theme, most participants reported having little to no knowledge of PrEP, highlighting the need for more comprehensive education efforts about its benefits for HIV prevention. In the second theme, the participants emphasized a need for more comprehensive education on sexual and reproductive health, encompassing topics like STIs, contraception, and sexual health check-ups. However, they also noted the seeming absence of active promotion of PrEP by healthcare providers, revealing a systemic gap in knowledge and information. Moreover, participants’ lack of meaningful sex conversations with parents further linked PrEP and STIs to broader education. Lastly, in the final theme, participants recalled varying awareness of the long-term impacts of STIs generally, and a strong belief in the effectiveness of PrEP as a prevention strategy. In the following sections, the authors detail these participants’ experiences, knowledge, and attitudes.

### 3.1. Black College Women’s Limited PrEP Knowledge

Across the focus groups, a consistent theme emerged: a significant lack of knowledge about PrEP among the participants. The statement from FGD 2, “Um, I don’t really know much about PrEP, but I remember, um, at school, they had like this free HIV testing thing, but I think’ it’s like a pill that you take like every four months,” serves as a representative example of the broader collective perspective within the groups. Participants across different groups had a common misunderstanding of PrEP, often confusing it with other HIV prevention methods like post-exposure prophylaxis (PEP). Indeed, participants mentioned the notion of taking a pill after potential HIV exposure to prevent acquisition, but admitted to having limited exposure to information or insight on the topic:

Honest[ly], I don’t know much. Is that the one where I think that—isn’t it, too, like the one where you like take the pill if you feel like you’ve been exposed or you could have possibly been exposed to HIV and it’s supposed to prevent you from getting it? But’ I’ll be honest, I don’t I don’t know much about it, never really looked into it.

(FGD 4)

The comments from FGD 4 further conveyed the prevailing limited understanding of PrEP among participants in the focus groups. This comment illustrates participants had a partial understanding of PrEP but lacked detailed knowledge about its usage and effectiveness. This misperception emphasizes the need for clear and accurate information dissemination, as well as distinguishing PrEP from other HIV prevention methods like PEP. In two of the FGDs, participants noted their knowledge of PrEP was primarily derived from TV commercials featuring an African American gay man who described his sexual experiences and subsequent PrEP benefits. They added, “It’s a commercial with, um, an African American gay man and, um, he talked about his sexual life, his sexual experience and how he uses PrEP and how that’s made it so much safer for him, and, yeah” (FGD 3). This reveals the pivotal role media has in influencing the perception of who PrEP can aid. However, a deeper analysis of these comments suggests the participants perceived a limitation in the scope of these commercials. Meaning, these commercials seemed to specifically target gay men and trans women, but it did not appear to cater to or address the needs and concerns of cisgender women, especially Black women.

In addition to confusion between PrEP and PEP among participants, there was also miscommunication and misunderstandings regarding the distinction between PrEP and antiretrovirals (ART). One participant added, “I think PrEP also is used for those–I might be wrong, but I think they’re also used for people who already have HIV to reduce their load to undetectable, so they can’t pass it.” (FGD 3). While it is true that the active ingredients in PrEP and ART align, they differ in clinical use. Correspondingly, it is incumbent upon researchers and community educators to provide more clear and accurate education and awareness efforts about the distinct purposes and applications of PrEP versus ART.

### 3.2. The Need for Comprehensive Sex Education: From Parents, Providers, and Beyond

Participants across various focus groups articulated a collective desire for comprehensive sexual and reproductive health education that promotes optimal wellness and encourages informed decision-making. Within this, there was collective recollection of a largely notable absence of meaningful conversations about sex with their parents, and an extension of this to healthcare providers who focused less on education and more on treatment (e.g., prescribing birth control pills/modalities rather than explaining family planning).

For the majority of participants, discussions about sex with their parents were either nonexistent or limited to rudimentary information such as condom usage, not getting pregnant, and pap smear testing. These conversations tended to be brief and did not encompass a broader understanding of sexual health like HIV prevention methods. This was exemplified in the second focus group discussion as a participant explained:

Yeah. I never really had that conversation with my parents, about having sex or anything. More so like use condoms, get your Pap smear, and [that’s] pretty much it and get us tested if we’re not- if I’m not in a relationship. Other than that, my parents would probably most likely tell me to abstain, and that’s that.

(FGD 2)

This limited communication between parents and their children about sexual health topics can have far-reaching implications, including knowledge gaps during their sexual maturation years, which include the onset of menarche and puberty. Moreover, this could contribute to discomfort with discussing HIV and PrEP-specific needs with healthcare providers, especially in instances where providers might lack PrEP awareness themselves.

The participants’ focus on a broader spectrum of reproductive and sexual health issues (e.g., sexually transmitted diseases (STDs), consent, pap smears, mammograms, birth control, abortion, and adoption), underscores why comprehensive sex education is necessary and must encompass HIV prevention. One participant exemplified this ideal, having stated:

I think it’s important for women to know about different, protective measures that you can take in partaking in sex. Like, you know, condoms, birth control. I think it’s important for women to know about abortion as an option or adoption as an option. Just kind of like what happen[s] after sex or what can happen. And then I think it’s also important for women to understand consent and getting consent or offering consent, having consent, stuff like that.

(FGD 3)

Within this comment and the ensuing discussion, there is an emphasis on the importance of comprehensive women’s wellness measures, such as mammograms and pap smears, for sexually active Black women, regardless of age. Participants viewed these screenings as integral components of their overall health and well-being, considering them a vital part of the broader HIV prevention discourse. This idea cuts across the focus groups as one participant in another group suggested, “women should better understand their body and how much they can handle before they start getting on birth control and having sex” (FGD 2). These comments suggest PrEP conversations should extend beyond their specific utility against HIV in favor of connecting them to a broader understanding of its medical implications within the context of overall sexual health.

Indeed, it is vital to link PrEP to women’s overall sexual wellness, because some participants described healthcare providers who seemed to prioritize discussions about condom use, pregnancy prevention, and birth control over PrEP. This was best exemplified in one participant’s comment: “I feel like, um, especially [among] women of color, … I feel like they talk more... more about like birth control, especially like birth control pills” (FGD 5). In other words, while these topics are undoubtedly important, there is a need for a more comprehensive approach to sexual health education and awareness that also centers PrEP. Taken together, these statements reflect the sentiment that sexual and reproductive health education should empower women with the knowledge of preventive measures like PrEP. Relatedly, they must also encompass a deeper understanding of their own bodies and their personal boundaries. These concurrent realities should compel providers to improve their knowledge about a range of sexual health interventions, thereby increasing overall harm reduction for both Black college women as individuals and the broader communities they exist within.

Nearly all participants had never heard of PrEP from their healthcare providers, consistent across four out of five FGDs. This led participants to express frustration and sighs of disappointment in their interactions with healthcare providers. In fact, one participant added, “I’ve never been offered PrEP by a healthcare provider” (FGD 1). While another recounted an experience where her obstetrician was unaware of PrEP when she brought it up during a visit. She explained, “Actually, I think I brought up PrEP to my, um, OB one time, and she didn’t like know what I was talking about. She was like, ‘Oh’ I’ve never heard of it” (FGD 2). This lack of awareness among healthcare professionals hinders PrEP and reveals inadequacy of healthcare provider education and training on PrEP. Ultimately, these findings emphasize the urgent need for healthcare provider education and training on PrEP, as well as a broader shift towards holistic sexual health discussions that encompass a range of prevention methods and considerations.

### 3.3. PrEP as an Effective Prevention Strategy

Across the focus group discussions, participants elucidated a comprehensive understanding of the general adverse consequences of STIs. Despite limited PrEP knowledge and awareness, participants highlighted taking proactive measures to protect their sexual health, responsible sexual practices, and informed decision-making beyond HIV. This is exemplified in the first group discussion, where a participant noted:

Even if you only have one partner, um, your one partner, they have multiple other partners and that’s where the danger—and that can come in and how it can spread. And, you know, it can affect you. Some STDs, if they go untreated, you can become infertile. So I feel like that’s a big part of it as well.

(FGD 1)

Here, participants underscore the critical point that even if an individual had only one sexual partner, their partner’s sexual history and behavior could significantly impact their own risk of contracting STIs. This awareness and interconnected nature of sexual health combine the consideration of not only one’s own behavior but also that of their sexual partners. Moreover, participants understood the potential gravity of untreated STIs, acknowledging some STIs could lead to irreversible effects, such as infertility.

With this in mind, participants advanced a common strategy of knowing ones’ partners’ STI test results before engaging in sexual intercourse. A participant explained:

I would say who they’re having like sexual interactions with other than you and how long they went to the doctor, like their previous checkup and if they’re STD positive or had any type of previous [history] with STDs.

(FGD 5)

This comment represents a collective sentiment among participants that commitment to making informed choices about their sexual health requires direct conversations with partners. This sort of information gathering suggests Black college women seek to engage in responsible sexual practices and informed decision-making, though such outcomes still heavily rely upon partner trust, honesty, and communication. And while overall knowledge of PrEP across this study was limited, participants’ awareness of the broader context of sexual health and its implications was evident.

Correspondingly, a consistent and unanimous agreement emerged regarding the effectiveness of PrEP in reducing the risk of HIV transmission. This is in part because the focus group discussions themselves served as a form of PrEP education and intervention. Participants eventually expressed a clear grasp of the purpose and modalities of PrEP, recognizing it as a medication that can significantly decrease the likelihood of contracting HIV. One participant noted:

You can take [it] daily, and it lessens your chance of getting, um, of contracting HIV. If you have frequent sexual partners or if you are in a relationship with somebody who is HIV positive. If you’re in an HIV discordant couple, then the person who is HIV negative can take it.

(FGD 2)

This collective understanding of PrEP efficacy as an HIV prevention method is a critical foundation for increasing its awareness and uptake among Black women. Moreover, some of the latter group discussions allowed dialogue to emphasize other notable benefits PrEP offers to women in serodiscordant relationships—or those who are sexually involved with partners living with HIV. These insights highlight the diverse contexts in which PrEP can play a crucial role in safeguarding women’s sexual health, including reducing the risk of HIV transmission in various relationship dynamics.

However, the emergence of corrective education within the focus groups—where participants exchanged knowledge, addressed misconceptions, and validated shared experiences—underscores how, in the absence of targeted studies and interventions, Black college women often become their own sources of health education and peer support. Some participants continued to repeat and reinforce the constraints around early PrEP guidance, which make it much more difficult to achieve greater PrEP uptake today. One participant explained,

… there was somehow some scarcity associated with it and, um, uh, I guess, a risk associated with it that only made it worth it if you were, um, you know, involved in a relationship with someone who was HIV positive.

(FGD 1)

Meaning, while there is growing understanding of PrEP’s value, the earliest PrEP guidelines focused and relied heavily on perceptions of risk and serodiscordant relationship status. While the science is clear that undetectable equals untransmittable (U = U), and the authors believe in this science, it is important for cisgender women to think about PrEP as a protective mechanism irrespective of a partner’s status. Indeed, the initial conceptions of who(m) PrEP is indicated for continue to shape how cisgender women conceive of whether PrEP is an intervention option for them.

Nevertheless, participants recognized PrEP could significantly reduce the chances of HIV transmission in these discordant relationships, providing an additional layer of protection for an HIV negative partner. This illuminates the potential of PrEP to contribute to safer and healthier relationships while partially protecting Black college women from acquiring HIV. Overall, these combined findings reveal how there is a demonstrated gap in general sexual health and PrEP education, suggesting that more nuanced, culturally relevant outreach efforts are needed to address the specific needs and concerns of women in various relationship dynamics to empower them to make informed decisions about their sexual health.

## 4. Discussion

### 4.1. Limited PrEP Awareness and Knowledge

Few studies have explored knowledge, awareness, and attitudes about PrEP among HBCU Black college women [13,19,27,28]. In the present study several themes emerged that revealed FGDs understanding and the factors that contributed to that understanding. For example, consistent with existing literature, the present findings reveal that HBCU Black college women in this study had limited to no knowledge of PrEP [28,48]. Among participants with limited knowledge, extensive misinterpretations were observed, including confusion between PrEP, PEP, and ART [28,36]. Through participation in the present study, participants went on to reveal understandings that PrEP minimizes the risk of acquiring HIV infection when engaging in unprotected sex, suggesting PrEP awareness is critical to more nuanced HIV prevention [34].

### 4.2. Contextual Influences on Awareness

Participants’ perspectives were also shaped by their positionality as college students navigating structured academic environments, where peer norms, institutional messaging, and evolving identity development collectively influenced their awareness, perceived risk, and engagement with HIV prevention efforts [53]. Meaning, a lack of PrEP awareness was common among the participants, reflecting broader structural and social determinants that impact Black college women understanding of HIV prevention [48]. These realities mirror broader discrepancies in PrEP awareness found outside the college context, further reflecting the need to focus on campus environments [10,12,13,16].

### 4.3. Perceptions of PrEP and HIV Prevention

While participants initially demonstrated gaps in knowledge, some recognized that PrEP was “an addition to condoms” and “offered hope to women and others at risk of HIV” [34]. These findings underscore the urgent need for comprehensive education and awareness campaigns on PrEP within the HBCU context and broader campus context generally [19,20]. Furthermore, these findings underscore how existing initiatives to promote PrEP are not effectively engaging these populations [17,21,23,38]. There is a need for clear and accessible information dissemination from not only healthcare providers, but also campus health professionals and leaders [40]. Such changes would ensure individuals are well-informed about the purpose, usage, and benefits of PrEP [29,31,32,34]. Additionally, participants’ responses reflected a limited understanding of PrEP, as participants demonstrated gaps in knowledge and expressed uncertainty about its uses [19,38].

Within the FGDs, there was a perceived lack of representation and awareness regarding the relevance and benefits of PrEP for women. This finding demonstrates the critical need for diversified and inclusive messaging in HIV prevention campaigns to ensure all populations with a greater need for PrEP, including cisgender women, are well-informed about PrEP as a viable prevention option [12,15]. Willie and their colleagues conducted an intersectional analysis in their study and found “gendered racism fuels the invisibility of Black women from PrEP marketing as a socio-structural barrier with the potential to delay PrEP initiation” (p. 6, [12]). Indeed, clear and targeted messaging can help Black women make informed decisions about their HIV prevention and treatment options while reducing the risk of misconceptions and misinformation [17].

### 4.4. Implications for Tailored Messaging and Interventions

Participants in two FGDs expressed the misconception that PrEP could also be used to reduce viral loads in individuals who were already living with HIV. One participant’s statement exemplified this confusion in theme one. While the active ingredients are similar or sometimes the same, ART is more complex than PrEP, and it is important to help the general public understand how these medications differ and how they are used for treatment and prevention, respectively. Moreover, the misinformation around ART raises concerns about the clarity of PrEP messaging. The findings concurrently revealed how several participants believed PrEP could effectively prevent the spread of HIV among discordant couples and was important for lowering HIV transmissions among persons living without HIV. In sum, this finding shows the confusion some Black women face when coming to know prevention and treatment medications. Nevertheless, participants highlighted how PrEP was particularly advantageous in relationships where one partner is living with HIV and the other is not. This suggests that the messaging around PrEP’s use in monogamous intimate partner relationships has generally been effective.

However, for Black women, like those in this study, who have multiple sexual partners and/or have had inconsistent condom usage, the messaging around the use of PrEP while engaging in casual sex may not be as effective. Moreover, this highlights why PrEP is a valuable resource for women who may be susceptible to HIV acquisition due to their partner’s HIV status [12]. While participants reported a range of relationship experiences–including closed, single and non-monogamous contexts– no consistent differences in perspectives or behaviors emerged based on relationship status, the relevance of PrEP and prevention messaging was broadly shared across participants.

Generally, the participants indicated healthcare providers had never offered them PrEP medications, thus suggesting a lack of adequate PrEP knowledge and awareness among providers. An unmet need for HIV prevention educational and awareness campaigns tailored for cisgender Black women should be considered. ViiV Healthcare’s Risk to Reason initiative boasts an innovative framework that centers on Black Women and includes guidance on messaging and marketing HIV prevention strategies [27]. Reshaping current cultural beliefs begins with honest conversations, acknowledging stigma, historical traumas, and the various structural and social determinants that affect both the community and the individual [27]. Moreover, taking a holistic approach to sexual and reproductive health education among Black women in college is necessary to combat the ways gendered racism renders this population invisible within intervention strategies. Scott et al. found that interventions should not address stigma from a one-size-fits-all model [39]. They suggested intersectional and cross-regional interventions should aim to reduce the sociocultural contextual influences that impact stigma, particularly amongst a non-monolithic population, like Black women. This approach should encompass a wide range of topics, from screenings and preventive measures to a deeper understanding of one’s own body and sexual health. Moreover, considering the nuanced and contextual ways stigma manifests for Black women is necessary to understanding how their knowledge and awareness of PrEP could translate to PrEP uptake [4].

### 4.5. Recommendations for Future Practice & Policy

Despite PrEP knowledge being low, participants’ interest and willingness to use PrEP if prescribed were high across the groups. The focus group findings highlight the need for improved communication between parents and their children about sexual health. Meaningful conversations that encompass a wider range of topics, including HIV prevention methods like PrEP, can better equip young women with the knowledge and resources needed to make informed decisions about their sexual health and reduce the risk of HIV transmission. Healthcare providers’ roles are significant in the low numbers of PrEP prescribed, due to provider bias or a lack of PrEP awareness [28]. Increasing PrEP training and awareness among healthcare providers, including primary care doctors, obstetricians/gynecologists, and emergency room physicians, will enhance the workforce for those prescribing PrEP [34,36]. National PrEP campaigns such as the CDC #ShesWell: PrEP for Women initiative are aimed at increasing PrEP awareness among women and healthcare providers, while the US Department of Health and Human Services Ready, Set, PrEP campaign is aimed at providing free PrEP for those who qualify [16,47]. Community-led interdisciplinary convenings, including the PrEP in Black America Summit and the “road map” for PrEP in Black communities, are established efforts aimed at addressing the needs of PrEP for Black women and Black America alike [46]. The findings reinforce the necessity of programs such as the ‘For Us by Us’ summit that translate community insights into actionable, equity-centered interventions.

### 4.6. Relevance of Geographic and Structural Context

Ultimately, these findings are especially significant considering many HBCU campuses, including the present study, are located within urban contexts with elevated HIV transmissions, or in highly rural communities with limited access to sexual healthcare providers [42,43]. In either context, PrEP education and awareness should more readily be available to Black college students who are among the leading subgroups of growing HIV transmissions. Indeed, additional focus on the U.S. South and other geographies of homespace where Black women and girls occupy require added focus [2,5,7,42]. Addressing the total well-being of Black women at HBCUs must include efforts to educate them on the benefits of PrEP and other STI/HIV prevention methods.

## 5. Limitations, Implications, and Concluding Thoughts

### 5.1. Study Parameters and Future Directions

Like all research studies, there are some parameters around this work [50,51,52]. Given that the current study involved topics such as HIV status, relationship status, communication regarding one’s range and number of sexual partners, and inconsistent condom usage, the likelihood of social desirability bias was high. This means participants may have offered responses that did not entirely reflect their safer sex praxis, either to avoid embarrassment or to present themselves more favorably. In addition, because confidentiality is more difficult to ensure in focus group settings, the potential for social desirability bias was heightened, which may have influenced the findings [50,51,52]. However, the lead author maintained that this approach was most useful given Black women’s unique communication practices, norms, and potential for opening up as evidenced by emerging qualitative approaches like sister circle methodology which suggest Black women in dialogue together yields a more culturally rich form of data [54]. Moreover, this study was conducted not long after the initial onset of the COVID-19 pandemic. Meaning, participants may have been less likely to disclose any sexual behaviors with a heightened potential for HIV and STI transmission because they would also convey a message about COVID-19 safety practices since the study was conducted during the pandemic.

Next, as a general qualitative inquiry, this study was much broader in scope than those using more specific methodological approaches (e.g., narrative, phenomenology, etc.) [50,51,52]. While this allowed the lead author to ask a broader range of questions and to be more flexible in the execution, it concurrently constrained the full spectrum and accuracy of participants’ responses. Lastly, while this research broadly examined students’ knowledge and awareness, the lead author could have considered the role of campus professionals and services in students’ development [20]. Meaning, future researchers exploring Black college women and PrEP at HBCUs should consider investigating campus healthcare provider attitudes, services, infrastructure, capacity, scheduling, etc. The authors also want to highlight that location matters [46,47]. Location is pivotal since the HBCU campus is located in a zip code that has both a high population of individuals living with HIV and is simultaneously designated as an EHE jurisdiction. This warrants a national examination to properly educate students. Thus, the institution needs additional resources to properly educate students and raise awareness about the critical role zip codes play.

### 5.2. Implications

This study illuminates several implications for the health policy and promotion professionals positioned to address the issues facing a lack of awareness of PrEP. Policymakers should consider strategies that increase knowledge of and access to PrEP medications, particularly for students at HBCUs. Less than half of HBCU health administrators reported any formal HIV prevention or intervention programming on their campus [37]. While some HBCUs have implemented programming to build awareness and promote health and well-being, local, state, and federal governments have halted dollars previously aimed at HIV prevention. Pitfalls in federal policy, specifically freezing grantmaking at the National Institutes of Health (NIH), create new challenges in the fight of increased awareness at HBCUs.

#### 5.2.1. Implications for Higher Education Leaders

This study reveals that higher education reproduces societal health inequities by ignoring the significance and urgency of HIV PrEP education. Higher education leaders at HBCUs should respond to the expressed desire by participants for comprehensive sexual and reproductive health education. The participants articulated their desire to engage in broad discussions surrounding HIV prevention and other sexual health-related topics. However, they currently lack access to relevant, accurate and complete information. Thus, it is imperative that higher education institutions provide these educational opportunities. The current attacks on NIH and equity-based research decrease the quality, quantity, and veracity of information available and accessible. By introducing students to broader and more culturally relevant sexual education in general, topics such as HIV and PrEP are not only discussed but also normalized. Thus, introducing the topics in orientation, carrying out marketing campaigns, and holding residence life events can be implemented to expose students to accurate sexual health education [20]. Ultimately, by making this information accessible, students can make informed decisions about their health.

#### 5.2.2. Implications for PrEP Campaign Activators, Planners, and Educators

These findings similarly have vital implications for future PrEP campaigns, especially those intentionally centering Black women. As trusted institutions within Black communities, HBCUs are uniquely positioned to deepen understanding around the perspectives, attitudes, and knowledge shaping PrEP uptake among their students [19]. Prioritizing this work—particularly with and for Black women—will be essential to advancing health equity and ensuring PrEP reaches those who need it most. Pairing the students’ perspectives with increased on-campus PrEP information, an on-campus PrEP provider, and normalizing sexual health conversations can further this study’s impact on the HBCU community. This approach will gain further insights and feedback for the influential social and structural determinants of health that impact college-age Black women’s daily lives [9,18].

#### 5.2.3. Implications for Clinical Care

Future public health efforts must consider actualizing individualized culturally appropriate frameworks, including combined evidence-based intervention approaches for PrEP delivery and uptake for and by Black women [27,47]. Within clinical care on college campuses, this means developing tailored communication strategies that directly address gaps in gaps, side-effect concerns, and misconceptions about who PrEP is “for.” Campus health providers should be trained to integrate PrEP counseling into routine reproductive and sexual health visits, ensuring confidentiality, trust, and a non-stigmatizing environment. Clinical interventions should also prioritize peer-led and community-based approaches that reflect the lived experiences of Black women, creating spaces where students feel represented in HIV prevention messaging [53]. Finally, partnerships between campus health centers and local public health agencies can expand access to PrEP and HIV testing, bridging systemic barriers and ensuring continuity of care beyond the college setting.

### 5.3. Conclusions

This qualitative study examined Black college women’s knowledge and awareness of PrEP within the context of HBCUs. Despite their longstanding role as central figures in sustaining communities, Black women continue to be overlooked in national HIV prevention strategies and persistently experience misunderstandings about PrEP’s purpose and availability. These knowledge gaps, particularly within HBCU contexts, highlight the need for continued research and efforts to improve PrEP awareness, knowledge and uptake among Black women until misconceptions and misperceptions are eliminated. Key findings reveal that while awareness of PrEP remains limited, students expressed openness to learning more when information was delivered through trusted peers and clinicians. Collectively, these findings hold important implications for the design of future PrEP initiatives—specifically those that center on Black women [19,20]. Ensuring equitable access, awareness, and uptake of PrEP for Black women must be recognized not as a peripheral concern but as a critical priority. Meaningful progress toward ending the HIV epidemic requires an intentional commitment to centering Black women’s experiences, needs, and voices in the design and implementation of prevention initiatives.

## Figures and Tables

**Table 1:** Demographic Characteristics of Focus Group Participants (*n* = 22).

	*N* (%)
Race	
African American/Black	22 (100%)
Age	
18–25	19 (86.4%)
26–35	2 (9%)
36–45	0 (0%)
46–55	1 (4.5%)
Sex	
Assigned female at birth	22 (100%)
Education level	
Freshman	10 (45.5%)
Sophomore	2 (9%)
Junior	6 (27.3%)
Senior	1 (4.5%)
Doctoral	3 (13.6%)
Relationship status	
Single	14 (63.6%)
In a relationship	8 (36.4%)
Married	1 (4.5%)
Multiple Sexual Partners	
Yes	3 (13.6%)
No	19 (86.4%)
Inconsistent Condom Usage	
Yes	11 (50%)
No	11 (50%)

## Data Availability

No publicly available data are available due to the intimate, qualitative nature of the present study. We closely guard the original data presented in this study to protect and maintain participant confidentiality.
